# Dynamic meiotic behavior and evolutionary insights of supernumerary B chromosomes in the hangingfly *Bittacus cirratus* (Mecoptera, Bittacidae)

**DOI:** 10.3897/compcytogen.19.153340

**Published:** 2025-06-04

**Authors:** Le-Le He, Ying Miao, He-Hong Wang, Jie Zhang, Bao-Zhen Hua

**Affiliations:** 1 Key Laboratory of Plant Protection Resources and Pest Management, Ministry of Education, College of Plant Protection, Northwest A&F University, Yangling, Shaanxi 712100, China Northwest A&F University Yangling China; 2 College of Agriculture, Ningxia University, Yinchuan, Ningxia 750021, China Ningxia University Yinchuan China

**Keywords:** Chromosome rearrangement, cytogenetics, heteromorphic homologues, karyotype, meiotic drive

## Abstract

Supernumerary B chromosomes are significant dispensable genetic elements that follow their own species-specific evolutionary pathways. Despite their widespread occurrence, comprehensive analyses of their meiotic behavior remain limited. In this study, we present the first systematic investigation of B chromosome morphology and meiotic behavior in the hangingfly *Bittacus cirratus* Tjeder, 1956 using cytogenetic approaches. The male basal chromosome numbers of *B. cirratus* is 2n = 30 + XO, with 0–5 polymorphic B chromosomes. Intraspecific B chromosome polymorphism manifests as various distinct morphotypes ranging from punctiform, bicentric, and ring-shaped to larger coiled forms, indicating that the B chromosomes may undergo rapid structural changes. During meiosis, B chromosomes display transmission drive through asymmetric segregation, preferentially accumulating in one daughter cell. Most B chromosomes formed univalents, with few forming bivalents or trivalents at meiosis I. Three unconventional retention mechanisms were identified in univalent B chromosomes: (1) associating with a nonhomologous chromosome, (2) accumulating near spindle poles, and (3) contributing to unequal spindle formation. Based on the abundant chromosomal changes of A chromosomes and stable XX/XO sex determination, we infer that the B chromosomes likely originated from multiple A chromosomes in *B. cirratus*. The roles of B chromosomes in the cell cycle and individual fitness are briefly discussed, and the evolutionary scenario is putatively put forward for the diversification of B chromosomes.

## ﻿Introduction

Supernumerary chromosomes (B chromosomes) are dispensable genetic elements of eukaryotic species (Beukeboom 1994; Ahmad and Martins 2019). They are considered byproducts of the standard karyotype evolution (Jones et al. 2008; Houben 2017; Pereira et al. 2017). Although numerous studies have pointed out that the effects of B chromosomes depend on their frequencies (Jones and Rees 1982; Jones and Houben 2003; Houben et al. 2014; Ahmad and Martins 2019), several studies indicate that they can also have biological functions, showing positive or adaptive effects (Pereira et al. 2017; Jones 2018; Langner et al. 2021; Ferree et al. 2024). Despite their recognized importance in genome manipulation and evolutionary processes (Camacho et al. 2000; Levin et al. 2005; Houben et al. 2014), little information is known about the detailed meiotic behavior and morphology of B chromosomes.

In many species, B chromosomes follow species-specific evolutionary pathways for deviating from usual Mendelian segregation (Houben 2017), often exhibiting transmission rates higher than the Mendelian expectation of 0.5, known as “chromosome drive” (Araújo et al. 2001; Birchler and Yang 2021; Ebrahimzadegan et al. 2023). B chromosomes do not pair or segregate during meiosis as A chromosomes do because of irregular meiotic behavior (Houben et al. 2013). B chromosomes are usually smaller than A chromosomes with variable morphologies ranging from punctiform to the smallest chromosomes of the regular set (Levin et al. 2005; Jones et al. 2008; Houben et al. 2013; Jang et al. 2016). B chromosomes exceed the size of A chromosomes only observed in a few species, such as the cyprinid fish *Alburnus alburnus* (Linnaeus, 1758) (Ziegler et al. 2003), the marsupial frog *Gastrotheca espeletia* Duellman et Hillis, 1987 (Schmid et al. 2002), and the grasshopper *Eyprepocnemis plorans* (Charpentier, 1825) (Bakkali and Camacho 2004). Although B chromosomes have attracted significant research interest over the years, studies across species remains largely uneven (Karafiátová et al. 2021).

More than 2800 eukaryotic species have been reported possessing B chromosomes, with insects being one of the most particularly frequent hosts (D’Ambrosio et al. 2017; Ahmad and Martins 2019). B chromosomes have been documented in various insects, including Archaeognatha (D’Ambrosio et al. 2017), Orthoptera (Hewitt 1974; Munoz et al. 1998; Warchałowska-Śliwa et al. 2020), Hemiptera (Nur 1966), Neuroptera (D’Ambrosio et al. 2017), Coleoptera (Xavier et al. 2018), Diptera (Hackstein et al. 1996), Lepidoptera (D’Ambrosio et al. 2017), and Hymenoptera (Araújo et al. 2001; Menezes et al. 2013). Mecoptera represents one of the most primitive orders in Holometabola (Beutel and Pohl 2006; Zheng et al. 2022; Ma et al. 2023), but up till now it has remained unknown whether B chromosomes exist in this order.

Bittacidae comprises approximately 220 extant species and is the only cosmopolitan family in Mecoptera (Byers and Thornhill 1983; Wang and Hua 2017). They are commonly known as hangingflies, because the adults have three pairs of raptorial legs, and hang on the edges of leaves or twigs of plants between flights (Byers 2002). Hangingflies are one of model organisms for studying mating behavior (Bornemissza 1966; Gao and Hua 2013; Wei et al. 2020) and are often regarded as ecological indicators (Byers and Thornhill 1983; Chen et al. 2013; Wang and Hua 2023). Cytogenetic data have been documented for seven species of Bittacidae, most of which exhibit distinct karyotypes and well-defined meiotic behavior (Matthey 1950; Atchley and Jackson 1970; Miao and Hua 2017, 2019, 2020). These characteristics suggest that hangingflies could be an excellent experimental material for studying chromosome biology.

In this study, we investigated the morphology and meiotic behavior of B chromosomes in detail in the hangingfly *Bittacus cirratus* Tjeder, 1956 toward a better understanding of this unique genetic element. Cytogenetic studies could shed light on the evolutionary mechanisms acting on chromosomes and provide reference for related research.

## ﻿Material and methods

### ﻿Insect sampling and rearing

**Figure 1.**
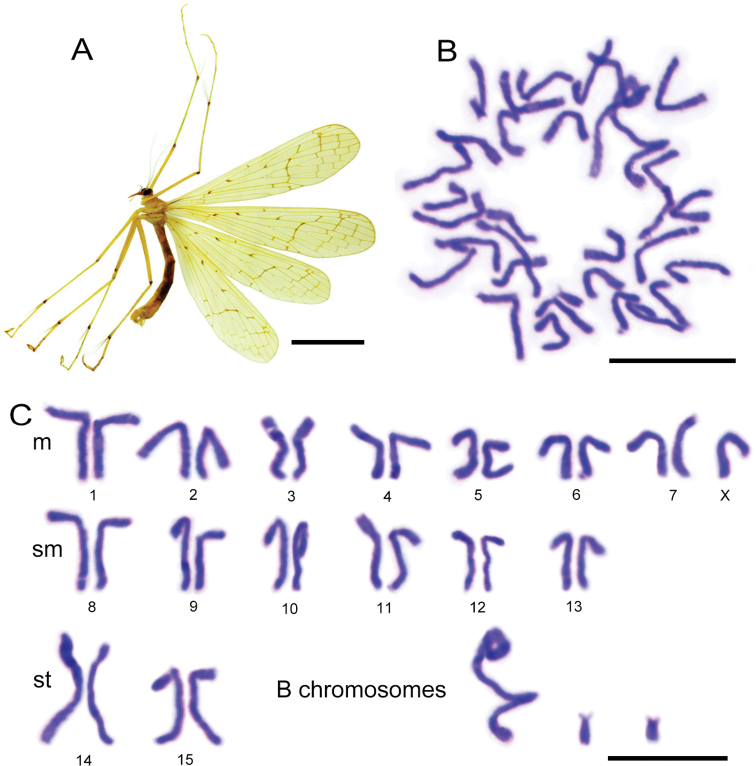
Habitus and mitotic karyogram of *Bittacus cirratus***A** male habitus in lateral view **B** mitotic metaphase came from the male testis of the fourth instar larva **C** karyogram showing 2*n* = 30 + XO + 3 B chromosomes. Abbreviations: m = metacentric; sm = submetacentric; st = subtelocentric. Scale bars: 5 mm (**A**); 10 μm (**B, C**).

Adults of *Bittacus cirratus* (Fig. 1A) were collected from the Qinling Mountains (34°13'48"N, 106°58'12"E, elevation 1450–1500 m), Shaanxi Province, China in August 2020. The live female adults were reared individually in plastic cups furnished with twigs and leaves of woody plants and moist absorbent cotton. The eggs, larvae, and pupae were incubated and reared in identical containers with damp humus. The adults and larvae were fed with freshly killed larvae of the yellow mealworm *Tenebrio molitor* Linnaeus, 1758 and frozen pupae of the house fly *Musca domestica* Linnaeus, 1758, respectively. Temperatures were maintained at 16 ± 2 °C for larvae, 21 ± 2 °C for pupae, and 23 ± 2 °C for adults. Relative humidity was maintained at 75% ± 10% (Miao and Hua 2017).

### ﻿Cytogenetic analyses

Chromosome spreads were prepared using the male testes of the third and fourth (last) instar larvae and pupae (Imai et al. 1988). C-banding (Giemsa staining) and fluorochrome DAPI (4’,6-diamidino-2-phenylindole) staining followed Miao and Hua (2019) and Rebagliati et al. (2003). Fluorescence signals were observed with a UV filter (330–385 nm), and photographs were captured with a Nikon DS-Fil digital camera mounted on a Nikon Eclipse 80i microscope (Nikon, Tokyo, Japan). The brightness and contrast of photographs were optimized using Adobe Photoshop 2022.

To analyze the sex determination mechanism, a total of 20 cells from 15 individuals at metaphase and anaphase of meiosis I were observed. Chiasma numbers were counted for 50 cells at diplotene. A total of 76 cells from 10 individuals were examined for counting diploid chromosome numbers at metaphase of mitosis and 100 cells from 20 individuals were examined for counting haploid chromosome numbers at meiotic metaphase II. Chromosomal morphology was determined based on the arm ratio, classifying chromosomes as metacentric (**m**), submetacentric (**sm**), subtelocentric (**st**), or telocentric (**t**) (Levan et al. 1964). Seven cells came from seven individuals with well-spread chromosomes at mitotic metaphase were measured. The following features of chromosomes were measured: long arm length (**L**), short arm length (**S**), relative chromosome length (**RL**) of each chromosome, arm ratio (*r* = *L/S*), and centromeric index (*i* = *S* × 100/*RL*). The captured images were quantified using the ImageJ software (https://imagej.net/).

## ﻿Results

### ﻿Polymorphic chromosomes

**Figure 2.**
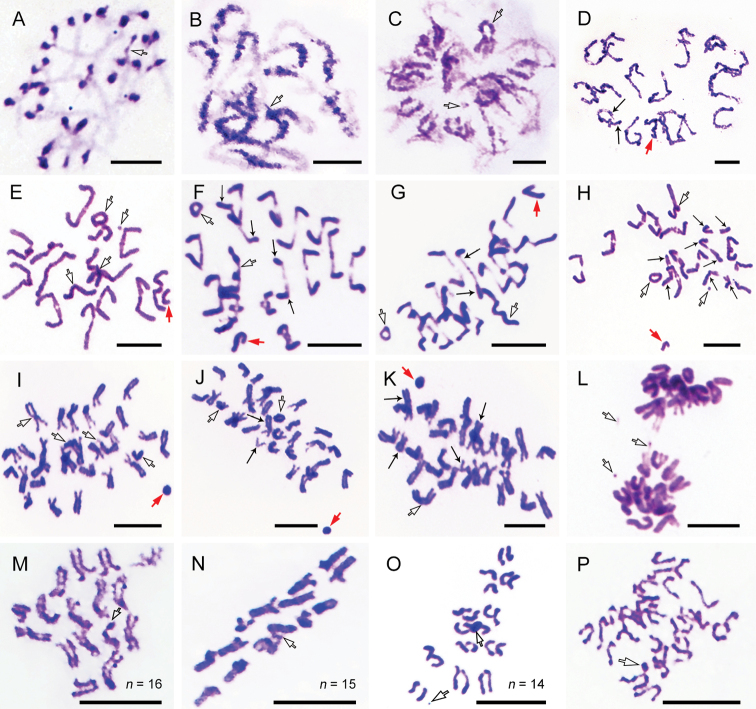
Meiosis of *Bittacus cirratus* stained by Giemsa **A** zygotene indicating homologues formed bivalents except univalent B chromosome **B** pachytene showing chromomeres linearly arranged on bivalents except B chromosome **C** diplotene showing dispersed synaptonemal complexes only crossed in exchanging sites, and B chromosome visible **D** diakinesis showing chiasmata at the end of chromosome arms, and centromeric regions formed knobs on bivalents **E–G** metaphase I showing bivalents assembled at the equatorial plate, multiple types of B chromosomes, and heteromorphic homologues **E** punctiform, ring-shaped, and large B chromosomes **F, G** B chromosomes formed trivalent **H–K** anaphase I showing bivalents separated into two independent chromosomes, and the sex chromosome moved undividedly towards one pole prematurely **H** one coiled B chromosomes **I** univalent B chromosomes stretched to one pole independently, and small B chromosomes attached to A chromosomes **J** the size of B chromosomes similar to A chromosomes **K** one bicentric B chromosome moved towards one pole prematurely **L** telophase I indicating punctiform B chromosome separated from sister chromatids, and small B chromosomes attached to a chromosomes **M–O** meiosis II showing variable haploid chromosome numbers **M** prophase II showing two sister chromatids separated from each other, but single B chromosome with nondisjunction **N** metaphase II showing the centromeres of the dyad chromosomes situated on the equatorial plate **O, P** anaphase II indicating B chromosomes not separated from sister chromatids. Open arrows indicate B chromosomes, red arrows show the sex chromosomes, and thin arrows point to heteromorphic homologues. Scale bars: 10 μm.

**Figure 3.**
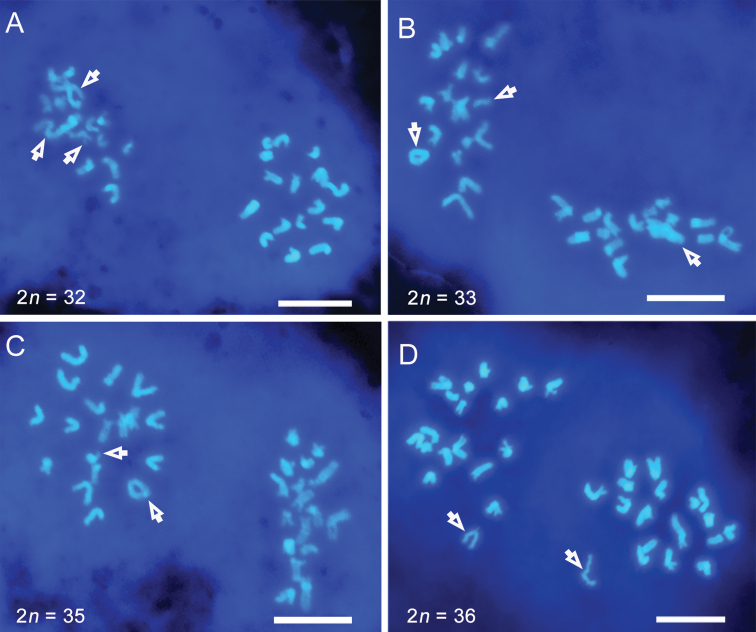
Meiotic metaphase II of *Bittacus cirratus* stained with DAPI **A–D** variable chromosome numbers and types of B chromosomes **A** large, coiled and punctiform B chromosomes, and one large B chromosome formed chiasmata with itself **B, C** ring-shaped B chromosomes **D** one B chromosome without sister chromatids prematurely transferred at meiosis II. Open arrows indicate B chromosomes. Scale bars: 10 μm.

Extensive chromosomal variation was observed in *Bittacus cirratus*. These polymorphisms result in the diploid number ranging from 2n = 30 to 36 (Table 1). A total of 34% cells possessed 31 chromosomes, and 19% contained 33 chromosomes. Haploid chromosome numbers ranged from n = 13 to 19, with n = 15 and 16 at high frequency of 36% and 30%, respectively. The karyotypes showed polymorphism 15–22 m + 6–15 sm + 0–4 st + 0–2 t (Fig. 1B, C, Suppl. material 1). The B chromosomes exhibited large morphological variation, and can be categorized into three groups: (1) larger than A chromosomes, (2) similar in size to A chromosomes, and (3) smaller than A chromosomes. Various types of B chromosomes were identified, including punctiform, ring-shaped, bicentric, and coiled forms (Figs 1C, 2, 3).

**Table 1.** Morphometric analyses and numerical statistics of chromosomes at mitosis metaphase of *Bittacus cirratus*.

m	sm	st	t	2*n*	Frequency	*n*	Frequency
22	6	0	2	30	0.09	13	0.02
15	12	4	0	31	0.34	14	0.03
16	12	4	0	32	0.16	15	0.36
16	12	4	1	33	0.19	16	0.30
16	13	4	1	34	0.07	17	0.21
16	15	4	0	35	0.04	18	0.06
19	15	2	0	36	0.11	19	0.02

Abbreviations: m = metacentric; sm = submetacentric; st = subtelocentric; t = telocentric; 2*n* = diploid chromosome numbers; *n* = haploid chromosome numbers.

### ﻿Meiotic characters

At early meiotic prophase I, the univalent B chromosomes were slenderer than the bivalents of A chromosomes (Fig. 2A, B). Although B chromosomes can also form bivalents, B chromosomes had no or one chiasma, fewer than the average 1.5 chiasmata per A chromosome at diplotene (Fig. 2C). The mean chiasma count per cell was 23.5 (ranging from 13 to 32). In addition, the sex chromosome formed a highly heteropycnotic body (Fig. 2D, F, G, I–K).

During metaphase I, all types of B chromosomes were observed (Fig. 2E–G). The meiotic behavior of univalent, bivalent, and trivalent B chromosomes was similar to that of the bivalents of A chromosomes, as they assembled at the equatorial plate and oriented towards the poles (Fig. 2F, G). Furthermore, one to four pairs of heteromorphic homologues were observed, with unequal-sized or different points of fiber attachment (Fig. 2F–H).

At anaphase I, the univalent sex chromosome shrank into vesical shape and moved undividedly towards one pole prematurely (Fig. 2H–K). Most B chromosomes were independently stretched towards one pole (Fig. 2H–K), including the bicentric B chromosome (Fig. 2K), yet one tiny B chromosome attached to the end of an A chromosome (Fig. 2I, L). The majority of ring-shaped B chromosomes were invisible (Fig. 2I–K), with a loss probability of around 70%. At telophase I, the B chromosomes underwent nondisjunction of sister chromatids except one punctiform B chromosome that separated into two B chromosomes (Fig. 2 L).

At meiosis II, variable chromosome numbers and multiple types of B chromosome were distinctly observed (Figs 2M–O, 3). The B chromosomes tended to aggregate in one of the two secondary spermatocytes (Fig. 3A–C), with a 60% probability that the chromosome number difference between the two daughter cells was at least two. A special event was observed that a large coiled B chromosome crossed over with itself (Fig. 3A). In addition, one metacentric B chromosome without sister chromatid moved prematurely towards one pole (Fig. 3D). Most B chromosomes were separated from sister chromatids at anaphase II, but a few B chromosomes moved to one pole with nondisjunction (Fig. 2P).

## ﻿Discussion

### ﻿Chromosomal variation

Extensive intra- and inter-individual variability for number and morphology of chromosomes were found in Mecoptera for the first time. Specifically, the diploid chromosome number ranged from 30 to 36 in *Bittacus cirratus*, primarily attributable to the presence of B chromosomes, with 2n = 30 + XO + 0–5 B chromosomes. The karyotypic diversity mainly arises from the notable differences in size and morphology of the heteromorphic homologues and B chromosomes. The presence of heteromorphic homologues likely results from chromosome rearrangements (Menezes et al. 2013; Xavier et al. 2018; Warchałowska-Śliwa et al. 2020), indicating recent or rapid evolution of A chromosomes.

The morphology of B chromosomes ranges from the smallest punctiform, ring-shaped, to the largest coiled forms within the genome. The emergence of punctiform B chromosomes is probably related to high number of chromosome rearrangements throughout the karyotype evolution (Houben et al. 2014; Xavier et al. 2018), and is reported as one of initial stages of B chromosomes development (Karamysheva et al. 2002). Freshly-broken ends of chromosomes are sticky and can join with any other such end (White 1957; Jackson 1971). If various types of extrachromosomal DNA sequences invade proto-B chromosomes, it leads to a larger size of B chromosomes (Rubtsov et al. 2004). This cumulative effect may be responsible for the formation of giant B chromosomes in *B. cirratus*. The addition of extensive repetitive sequences to sticky ends facilitates the formation of ring-shaped B chromosomes (Cohen et al. 2008; Hollis et al. 2020). In addition, coiled B chromosomes forming chiasmata during meiosis indicate that the B chromosomes may consist of homologous chromosomal fragments. The high degree variation of B chromosomes suggests that they may undergo a number of rapid structural changes and have different evolution processes.

### ﻿Meiotic behavior of B chromosomes

The B chromosomes did not pair or segregate during meiosis as the A chromosomes in *B. cirratus*, indicating that B chromosomes follow non-Mendelian inheritance, such as that reported in the beetle *Euchroma gigantea* (Linnaeus, 1758) (Xavier et al. 2018) and the wasp *Metapolybia decorata* (Gribodo, 1896) (Menezes et al. 2013). Most B chromosomes formed univalents, with few forming bivalents or trivalents at meiosis I. Bivalent B chromosomes separate normally, which is an important mechanism to maintain B chromosomes segregation and accumulation (Houben et al. 2014). However, univalents and trivalents gave irregular chromosome segregations so that there was a certain level of meiotic elimination. Univalent B chromosomes were the predominant form during meiosis, with a few being lost at anaphase I. We propose that some univalent B chromosomes may be too short to receive sufficient spindle fibers to properly segregate, leading to their loss, similar to the behavior of univalent B chromosomes reported in *Allium cernuum* Roth, 1798 (Grun 1959). Interestingly, some univalent B chromosomes had the capacity to move to one of the daughter nuclei and separate at anaphase II. There are three scenarios in which univalent B chromosomes can escape loss during meiosis.

One way to prevent univalent B chromosomes from being lost is to incorporate undivided univalent B chromosomes into the telophase I nuclei by associating a univalent B with a nonhomologous chromosome, as reported in the groundhopper *Tetrix ceperoi* (Bolívar, 1887) (Henderson 1961). This situation is in accordance with our observation, in which one tiny univalent B chromosome that formed a quasi-bivalent with A chromosomes moved normally to opposite poles during anaphase I (Fig. 2I). Another way is that the univalent B chromosomes accumulate near one of the two spindle poles and consequently remain in one of the telophase nuclei after anaphase I, for example in the plant *Plantago serraria* Linnaeus, 1758 (Catcheside 1950). This behavior was observed in the sex chromosomes and some B chromosomes during meiosis I in our study (Fig. 2K). Additionally, we found that most univalent or independent B chromosomes escaping loss during meiosis had a relatively large size. Combined with the fact that asymmetrical spindles are key components of the meiotic drive of the B chromosomes (Birchler and Yang 2021), we tentatively put forward a hypothesis that large B chromosomes may be resistant to the meiotic loss by contributing to unequal spindle formation for providing higher pulling force on the B centromere towards the generative pole.

B chromosomes were aggregated in one of the daughter cells, indicating that the B chromosomes transmission was significantly higher than the Mendelian expectation, performing drive (Araújo et al. 2001; Houben et al. 2014), as in maize (Lamb et al. 2006). This asymmetry of meiotic division enables the maintenance and accumulation of B chromosomes in the next generation (Birchler and Yang 2021). This might explain why the number of B chromosomes in hangingflies can accumulate to a relatively high number, maximally reaching up to five.

### ﻿The evolutionary scenario of B chromosomes

**Figure 4.**
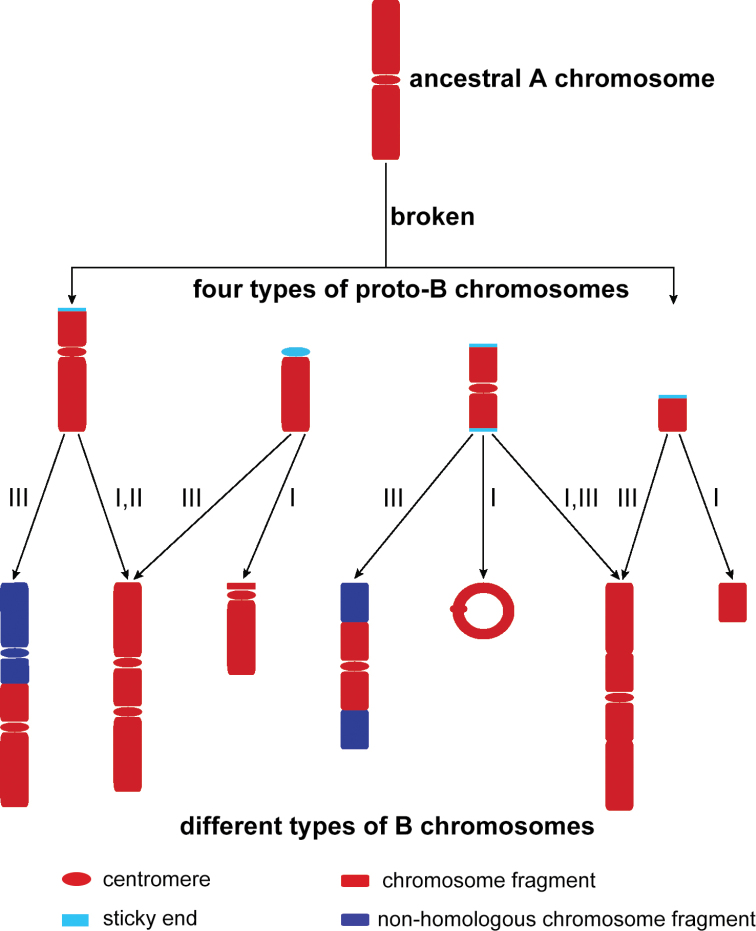
A schematic diagram for the formation of multiple types of B chromosomes in *Bittacus cirratus*, showing that ancestral A chromosomes fragmented into four types of proto-B chromosomes with sticky ends. Various chromosome rearrangement events, such as duplication (I), inversion (II), and translocation (III), contribute to the development of distinct types of B chromosomes, including bicentric, punctiform, ring-shaped, and large forms.

There is no general ubiquitous mechanism for the evolutionary origin of B chromosomes. In most species, B chromosomes originated from the standard complement, either A chromosomes or sex chromosomes (Camacho et al. 2000; Levin et al. 2005; Houben 2017; Ahmad and Martins 2019). According to the abundant chromosomal rearrangements observed in A chromosomes and stable sex chromosomes as ancestral character (Miao and Hua 2020), we suggest that the B chromosomes are derived from multiple A chromosomes, and put forward an evolutionary scenario for the diversification of B chromosomes in *B. cirratus* (Fig. 4).

Initially, ancestral A chromosomes break into different types of proto-B chromosomes with sticky ends at broken points (White 1957; Jackson 1971). Proto-B chromosomes fuse with each other, add DNA sequences, or duplicate to give stability and isolation from recombination (Camacho et al. 2000), which is considered as the starting point for the independent evolution of B chromosomes (Banaei-Moghaddam et al. 2015). They continue to undergo chromosomal duplication, inversion, or translocation accompanied by changes that would determine the final B size and morphology. B chromosomes without a centromere will be eliminated eventually (Martis et al. 2012).

### ﻿Cell cycle and biological effects

Notably, *B. cirratus* exhibits a large variation in chromosome number, a higher number and frequency of chiasmata, and more complicated morphology and behavior of chromosomes, which markedly differ from the stable karyotypes and conventional behaviors reported in other hangingflies (Matthey 1950; Atchley and Jackson 1970; Miao and Hua 2017, 2019, 2020). The presence of B chromosomes may alter the frequency and distribution of chiasmata, cause breakage of autosomes, and disturb the processes of meiosis, which have been documented in many organisms (Rhoades et al. 1967; Ayonoadu and Rees 1968; Jones and Rees 1969; Evans and Davies 1985; Cohen et al. 2008; Boroń et al. 2020), implying their key roles in the cell cycle and chromosome-related processes within the cell.

Both heteromorphic homologues and B chromosomes reflect highly chromosomal rearrangements and variations in *B. cirratus*. Such chromosomal variations may confer varying fitness by generating novel character states to favor natural selection (Dyer 1979; Coghlan et al. 2005; Faria and Navarro 2010; Gould et al. 2017; Cardoso and Cristiano 2021). Most hangingflies have restricted geographical distribution (Byers and Thornhill 1983; Chen et al. 2013), whereas *B. cirratus* is widely distributed from Russia to China (Tjeder 1956; Chen et al. 2013; Savitsky and Timokhov 2021). In fact, B chromosomes are often observed in species with wider geographic or host ranges (Jones and Puertas 1993; Komluski et al. 2022). This chromosomal diversity likely contributes to the individual fitness of *B. cirratus*. However, further validation through biogeographical and cytogenetic studies across different populations of *B. cirratus* is essential to elucidate the functional significance of these chromosomal features.

## Supplementary materials

Supplementary material 1 Morphometric analyses of the chromosomes of *Bittacus cirratus* based on seven spermatogonial cells Data type xls https://binary.pensoft.net/file/1345264This dataset is made available under the Open Database License
(http://opendatacommons.org/licenses/odbl/1.0/). The Open Database License (ODbL) is a
license agreement intended to allow users to freely share, modify, and use this
Dataset while maintaining this same freedom for others, provided that the original
source and author(s) are credited.Le-Le He, Ying Miao, He-Hong Wang, Jie Zhang, Bao-Zhen Hua
